# An exploratory qualitative study describing frontline nurses’ experiences with Presenteeism due to the COVID-19 pandemic

**DOI:** 10.1192/j.eurpsy.2022.1325

**Published:** 2022-09-01

**Authors:** C. Laranjeira, A. Querido

**Affiliations:** Polytechnic of Leiria, School Of Health Sciences/ Citechcare, Leiria, Portugal

**Keywords:** Pandemics, Workplace, Nurses, presenteeism

## Abstract

**Introduction:**

The COVID-19 pandemic will have a long-lasting impact on healthcare workplaces and professionals alike. For that reason, it is necessary more knowledge and insights about sickness presenteeism behaviour to provide appropriate occupational health services for all healthcare workers affected directly and indirectly by this pandemic.

**Objectives:**

The aim of this study was to explore and describe presenteeism experiences among frontline nurses due to the COVID-19 pandemic.

**Methods:**

A qualitative thematic analysis was used to evaluate the perceptions of frontline nurses from different Portuguese hospital institutions joined in two Focus Groups. Using convenience sampling a total of 20 RNs participated in interviews. No restriction was given to their gender, age, career, and wards in charge so as to obtain diverse data on nurses’ experiences of presenteeism.

**Results:**

The sample mean age was 36 years [range 25 - 42 years]; they had a clinical career of 12 years on average [range 2 - 20 years]. The major theme was the metaphor of “the rotten orange”. This theme implied the presence of a phenomenon that is invisible due to the ignorance of many, but which spreads through the members of a team, leading to an overload of its members for lack of one compassionate leadership. Consequently, leads to loss of the nursing spirit and nursing manpower.

**Conclusions:**

Our findings point to the development of workplace interventions targets to reduce healthcare worker presenteeism and to help employers foster a ‘healthier’ sickness culture during the pandemic and beyond.

**Disclosure:**

No significant relationships.

